# Cohort Profile Update: The Northern Ireland Longitudinal Study (NILS)

**DOI:** 10.1093/ije/dyaf054

**Published:** 2025-05-23

**Authors:** Estelle Lowry, Ian Shuttleworth, Peter Wilgar, Catherine McLoughlin, Emma Connell

**Affiliations:** School of Natural and Built Environment, Queen’s University Belfast, Belfast, UK; School of Natural and Built Environment, Queen’s University Belfast, Belfast, UK; Northern Ireland Statistics and Research Agency, Belfast, UK; Northern Ireland Statistics and Research Agency, Belfast, UK; Northern Ireland Statistics and Research Agency, Belfast, UK

**Keywords:** administrative data, longitudinal data, Northern Ireland, health, policy, population

Key Features

- The Northern Ireland Longitudinal Study (NILS)—a linked, administrative dataset comprising ∼28% of the resident population in Northern Ireland was established in 2006 to fulfill a range of academic and policy-research purposes.
- A further four census linkages have enabled a lifespan of >40 years for new research on health transitions, migration, social mobility, and other population trends.
- The most recent census linkage in 2021 comprised a total cohort of 515 930 people in the age range of 0 to 100+ years.
- The new linkage of environmental and school datasets in addition to the latest census provides the opportunity for new topics of research including self-reported chronic health conditions, national identity and language, sexual orientation, and renewable energy.
- The NILS has already been used by many researchers in academic and non-academic organizations. For new projects and enquiries, please contact nils@qub.ac.uk.

## The original cohort

The Northern Ireland Longitudinal Study (NILS) is an extensive system that integrates various administrative datasets, including census data, by linking them through medical-card registrations. This results in a study cohort of ∼515 930 (NILS 2021), representing ∼28% of the medical-card registrations. A detailed description of the linkage process and how it was established is available in the original cohort profile by O’Reilly *et al*. [1]. Technical protocols are also available on the website at https://www.nisra.gov.uk/support/research-support/northern-ireland-longitudinal-study-nils for each linkage of census data.

A related initiative, the Northern Ireland Mortality Study (NIMS), is built around the complete census-enumerated population (1 872 257 people in NIMS 2021). In this study, death registrations are connected to the census data by using a linkage process that is similar to that used for the NILS [1].

The establishment of both the NILS and the NIMS was initially funded by the Department of Health, Social Services and Public Safety, alongside the Research and Development Office of the then Health and Personal Social. Following their launch in 2006, the funding model has evolved slightly over the years and the ongoing support for development and maintenance is via the Health and Social Care Research and Development Division of the Public Health Agency, and the Economic Social and Research Council (ESRC). The Northern Ireland Statistics and Research Agency (NISRA) also provides financial assistance through the provision of data linkage via the Trusted Third Party, accommodation to house all aspects of the NILS/NIMS infrastructure, and staff to maintain and develop the databases. The resource is managed in cooperation with the academic director and co-investigators based in Queen’s University Belfast (QUB).

## What is the reason for the new focus?

QUB and NISRA work together to provide a service that goes beyond simply managing the data. In the past 5 years, the Research Support Unit (RSU) have utilized a stakeholder-engaged collective intelligence approach to inform the transformation of the NILS–RSU infrastructure [2]. The RSU have a clear commitment to meeting core user needs and part of this remit is to explore and create new data linkages, support the accessibility of data, and research outputs.

Since the publication of the original cohort profile, the dataset has expanded in both duration and breadth; four additional censuses were linked (a partial 1981 link with selected variables only, 1991, 2011, and 2021) and supplementary funding also enabled the linkage of historical vital-events data. The NILS now spans over 40 years, enabling a more complete depiction of the lives of Northern Ireland residents through the transition from conflict to peace.

Much of the new focus arises from questions asked in the census for the first or second time. The census has adapted its questions to meet evolving societal needs. As part of the 2021 Census consultation, NISRA held a series of topic consultation events in October 2015 to engage various stakeholders, including political leaders, advisory groups, public bodies, senior officials within government departments, and academic key users. The consultation process encouraged public participation to ensure inclusivity in designing the 2021 Census. These events allowed participants to seek clarification, provide feedback, and debate the topics included in the census. Participants gave comments on the need for specific questions given user needs, the census—and hence the NILS’—output geography, continuity with previous censuses, and the potential for using alternative data sources.

## What will be the new areas of research?

Due to the rich data in the NILS and its large, representative sample, it offers vast opportunities for research on many topics. The NILS provides an ideal mechanism for understanding the population health dynamics of Northern Ireland because of its wealth of demographic, individual socioeconomic, and household variables. Since the original cohort profile, school and environmental data have been added, providing for new research areas on pollution [3] and schooling [4]. Furthermore, the linkage of three new censuses (1991, 2011, and 2021) plus selected variables within the 1981 Census have added more variables with new and subsequent questions on self-reported chronic health conditions, national identity and language, sexual orientation, and renewable energy. Tables 1–3 provide an overview of NILS and NIMS member characteristics in comparison with the full census data and the observed trends highlight some potential areas of interest. We observe an aging population with increases in the age category of ≥65 years, housing tenure shows considerably higher proportions of shared ownership in later years, and the proportion of those holding a UK passport had only decreased at the time of the most recent census. The number of people stating a long-term health condition has also increased, particularly noticeable in those stating a breathing, emotional, or mental health condition. Characteristics tables spanning back to 1991 can also be accessed on our website (http://www.nils.ac.uk).

**Table 1.** Demographic characteristics of the NILS and the NIMS members compared with corresponding census years.

In cohort (number)	2011 Cohort			2021 Cohort		
	**NILS, *n* (%)** 486 677	**NIMS, *n* (%)** 1 744 461	**Census, *n* (%)** 1 835 184	**NILS, *n* (%)** 515 930	**NIMS, *n* (%)** 1 872 257	**Census, *n* (%)** 1 903 175
**Age (years)**						
0–15	100 666 (20.7)	356 937 (20.5)	380 758 (20.7)	106 440 (20.6)	383 047 (20.5)	388 433 (20.4)
16–34	123 866 (25.5)	457 099 (26.2)	493 938 (26.9)	117 382 (22.8)	431 497 (23.0)	443 828 (23.3)
35–64	188 964 (38.8)	671 391 (38.5)	696 699 (38.0)	202 153 (39.2)	734 521 (39.2)	744 438 (39.1)
65–74	40 363 (8.3)	142 919 (8.2)	145 642 (7.9)	48 811 (9.5)	175 144 (9.4)	176 932 (9.3)
≥75	32 818 (6.7)	116 115 (6.7)	118 147 (6.4)	41 144 (8.0)	148 048 (7.9)	149 544 (7.9)
**Sex**						
Male	235 719 (48.4)	846 664 (48.5)	898 611 (49.0)	251 638 (48.8)	917 879 (49.0)	936 129 (49.2)
Female	250 958 (51.6)	897 797 (51.5)	936 573 (51.0)	264 292 (51.2)	954 378 (51.0)	967 046 (50.8)
**Marital status (≥17 years)**	378 978	1 363 586	1 427 316	402 829	1 466 136	1 491 168
Married or in a civil partnership	187 067 (49.4)	663 067 (48.6)	681 374 (47.7)	189 722 (47.1)	686 430 (46.8)	693 236 (46.5)
Never married	128 997 (34.0)	475 806 (34.9)	513 758 (36.0)	146 449 (36.4)	538 646 (36.7)	553 152 (37.1)
Separated/widowed/divorced	62 914 (16.6)	224 713 (16.5)	232 184 (16.3)	66 658 (16.5)	241 060 (16.4)	244 780 (16.4)
**Housing tenure**						
Owner	351 615 (72.2)	1 251 953 (71.8)	1 294 856 (70.6)	356 186 (69.0)	1 279 654 (68.3)	1 293 565 (68.0)
Shared ownership	2806 (0.6)	9779 (0.6)	10 112 (0.6)	65 829 (12.8)	238 429 (12.7)	243 911 (12.8)
Private renter	119 018 (24.5)	432 503 (24.8)	475 666 (25.9)	88 282 (17.1)	327 826 (17.5)	339 351 (17.8)
Other^a^	13 238 (2.7)	50 226 (2.9)	54 550 (3.0)	5633 (1.1)	26 348 (1.4)	26 348 (1.4)

a Other category refers to unassigned responses including those residing in communal establishments.

**Table 2.** Information relating to identity of the NILS and NIMS members compared with their corresponding census year.

In cohort (number)	2011 Cohort			2021 Cohort		
	**NILS, *n* (%)** 486 677	**NIMS, *n* (%)** 1 744 461	**Census, *n* (%)** 1 835 184	**NILS, *n* (%)** 515 930	**NIMS, *n* (%)** 1 872 257	**Census, *n* (%)** 1 903 175
**Sexual identity** ^a^ **(≥16 years)**				**409** **490**		
Straight or heterosexual				371 194 (71.9)	1 342 491 (71.7)	1 363 858 (71.7)
Gay, lesbian, bisexual, other sexual orientation				8216 (1.6)	30 093 (1.6)	31 616 (1.7)
Prefer not to say or not stated				30 080 (5.8)	116 626 (6.2)	119 268 (6.3)
No code required				106 440 (20.6)	383 047 (20.5)	388 433 (20.4)
**National identity** ^b^						
British only				165 910 (32.2)	598 613 (32.0)	606 263 (31.9)
Irish only				150 191 (29.1)	544 491 (29.1)	554 415 (29.1)
Northern Irish only	100 977 (20.7)	358 355 (20.5)	379 291 (20.7)	102 829 (19.9)	369 878 (19.8)	376 444 (19.8)
British and Irish only				3153 (0.6)	11 506 (0.6)	11 766 (0.6)
British and Northern Irish only	30 384 (6.2)	107 827 (6.2)	111 757 (6.1)	41 945 (8.1)	149 080 (8.0)	151 327 (8.0)
Irish and Northern Irish only	4911 (1.0)	17 863 (1.0)	19 132 (1.0)	9041 (1.8)	32 800 (1.8)	33 581 (1.8)
British, Irish, and Northern Irish only				7685 (1.5)	27 509 (1.5)	28 051 (1.5)
Other	350 405 (72.0)	1 260 416 (72.3)	1 325 004 (72.2)	35 176 (6.8)	138 380 (7.4)	141 328 (7.4)
**Passport held**						
UK only	277 654 (57.1)	990 536 (56.8)	1 035 545 (56.4)	242 440 (47.0)	874 731 (46.7)	887 655 (46.6)
Ireland only	91 228 (18.7)	322 541 (18.5)	343 234 (18.7)	137 427 (26.6)	495 773 (26.4)	504 526 (26.5)
UK and Ireland only	8054 (1.7)	28 934 (1.7)	30 282 (1.7)	28 599 (5.5)	102 771 (5.5)	104 475 (5.5)
UK and other (not Ireland)	1101 (0.2)	4020 (0.2)	4310 (0.2)	1724 (0.3)	6343 (0.3)	6528 (0.3)
Ireland and other (not UK)	592 (0.1)	2092 (0.1)	2308 (0.1)	973 (0.2)	3611 (0.2)	3701 (0.2)
UK, Ireland, and other	108 (0.02)	363 (0.02)	382 (0.02)	452 (0.1)	1496 (0.1)	1549 (0.1)
Other only (not UK or Ireland)	13 322 (2.7)	50 358 (2.9)	55 805 (3.0)	22 637 (4.4)	90 549 (4.8)	92 548 (4.9)
No passport	94 618 (19.4)	345 617 (19.8)	363 318 (19.8)	81 678 (15.8)	296 983 (15.9)	302 193 (15.9)
**Religion^c^**						
Catholic	216 744 (44.5)	770 780 (44.2)	818 515 (44.6)	236 416 (45.8)	858 610 (45.9)	869 754 (45.7)
Protestant and other Christian (including Christian-related)	237 535 (48.8)	845 479 (48.5)	876 110 (47.7)	227 288 (44.1)	816 798 (43.6)	827 545 (43.5)
Other religions	4119 (0.9)	15 694 (0.9)	16 954 (0.9)	6811 (1.3)	26 989 (1.4)	28 515 (1.5)
None	28 279 (5.8)	112 508 (6.4)	123 605 (6.7)	45 415 (8.8)	169 860 (9.1)	177 361 (9.3)

a New question for the 2021 Census.

b New categories of response for the 2021 Census.

c Where “religion belongs to” was missing, response to “religion brought up in” was used.

**Table 3.** Information relating to health of the NILS and NIMS members compared with their corresponding census year.

	2011 Cohort			2021 Cohort		
	**NILS, *n* (%)** 486 677	**NIMS, *n* (%)** 1 744 461	**2011 Census, *n* (%)** 1 835 184	**NILS, *n* (%)** 515 930	**NIMS, *n* (%)** 1 872 257	**2021 Census, *n* (%)** 1 903 175
**General health**						
Very good	227 127 (46.7)	812 808 (46.6)	865 635 (47.2)	256 728 (49.8)	934 949 (49.9)	951 126 (50.0)
Good	154 415 (31.7)	550 138 (31.5)	576 532 (31.4)	147 881 (28.7)	537 458 (28.7)	546 033 (28.7)
Fair	73 611 (15.1)	260 652 (14.9)	269 078 (14.7)	71 244 (13.8)	256 153 (13.7)	259 983 (13.7)
Bad	22 066 4.5)	78 178 (4.5)	80 562 (4.4)	30 006 (5.9)	107 231 (5.7)	108 962 (5.7)
Very bad	5842 (1.2)	20 899 (1.2)	21 591 (1.2)	10 071 (2.0)	36 466 (1.9)	37 071 (1.9)
**Health conditions**						
**Autism or Asperger’s Syndrome** ^a^						
Has				9517 (1.8)	34 654 (1.9)	35 367 (1.9)
Does not have				506 413 (98.2)	1 837 603 (98.1)	1 867 808 (98.1)
**Breathing**						
Has	42 872 (8.8)	151 793 (8.7)	157 958 (8.6)	53 511 (11.6)	192 714 (10.3)	195 755 (10.3)
Does not have	440 189 (90.4)	1 570 882 (90.0)	1 655 440 (90.2)	462 419 (89.6)	1 679 543 (89.7)	1 707 420 (89.7)
**Communication difficulty** ^b^						
Has	7850 (1.6)	28 216 (1.6)	29 891 (1.6)			
Does not have	475 211 (97.6)	1 694 459 (97.1)	1 783 507 (97.2)			
**Emotional or mental health** ^c^						
Has	28 384 (5.8)	101 696 (5.8)	105 597 (5.8)	45 160 (8.8)	161 839 (8.6)	165 130 (8.7)
Does not have	454 677 (93.4)	1 620 979 (92.9)	1 707 801 (93.1)	470 770 (91.2)	1 710 418 (91.4)	1 738 045 (91.3)
**Hearing**						
Has	25 629 (5.3)	90 903 (5.2)	93 111 (5.1)	29 843 (5.8)	108 179 (5.8)	109 459 (5.8)
Does not have	457 432 (94.0)	1 631 772 (93.5)	1 720 287 (93.7)	486 087 (94.2)	1 764 078 (94.2)	1 793 716 (94.2)
**Intellectual or learning disability** ^a^						
Has				4544 (0.9)	16 597 (0.9)	16 921 (0.9)
Does not have				511 386 (99.1)	1 855 660 (99.1)	1 886 254 (99.1)
**Learning difficulty**						
Has	10 496 (2.2)	37 294 (2.1)	40 201 (2.2)	16 081 (3.1)	58 592 (3.1)	59 889 (3.2)
Does not have	472 565 (97.1)	1 685 381 (96.6)	1 773 197 (96.6)	499 849 (96.9)	1 813 665 (96.9)	1 843 286 (96.9)
**Memory**						
Has	9602 (2.0)	34 571 (2.0)	35 620 (1.9)	10 075 (2.0)	37 186 (2.0)	37 789 (2.0)
Does not have	473 459 (97.3)	1 688 104 (96.8)	1 777 778 (96.9)	505 855 (98.0)	1 835 071 (98.0)	1 865 386 (98.0)
**Mobility—limits to physical activity**						
Has	57 073 (11.7)	201 919 (11.6)	207 208 (11.3)	57 459 (11.1)	204 853 (10.9)	207 588 (10.9)
Does not have	425 988 (87.5)	1 520 756 (87.2)	1 606 190 (87.5)	458 471 (88.9)	1 667 404 (89.1)	1 695 587 (89.1)
**Mobility—requires wheelchair** ^a^						
Has				7636 (1.5)	27 773 (1.5)	28 136 (1.5)
Does not have				508 294 (98.5)	1 844 484 (98.5)	1 875 039 (98.5)
**Other**						
Has	25 583 (5.3)	91 309 (5.2)	94 660 (5.2)	46 229 (9.0)	165 615 (8.8)	167 751 (8.8)
Does not have	457 478 (94.0)	1 631 366 (93.5)	1 718 738 (93.7)	469 701 (91.0)	1 706 642 (91.2)	1 735 424 (91.2)
**Pain**						
Has	50 689 (10.4)	178 227 (10.2)	182 858 (10.0)	61 257 (11.9)	217 391 (11.6)	220 331 (11.6)
Does not have	432 372 (88.8)	1 544 448 (88.5)	1 630 540 (88.8)	454 673 (88.1)	1 654 866 (88.4)	1 682 844 (88.4)
**Sight**						
Has	8349 (1.7)	30 010 (1.7)	30 873 (1.7)	9269 (1.8)	33 467 (1.8)	33 957 (1.8)
Does not have	474 712 (97.5)	1 692 665 (97.0)	1 782 525 (97.1)	506 661 (98.2)	1 838 790 (98.2)	1 869 218 (98.2)
**One or more types of long-term health conditions**						
No health conditions	328 038 (67.4)	1 173 145 (67.2)	1 244 038 (67.8)	335 243 (65.0)	1 222 568 (65.3)	1 243 370 (65.3)
One or more health conditions	155 023 (31.9)	549 530 (31.5)	569 360 (31.0)	180 687 (35.0)	649 689 (34.7)	659 805 (34.7)

a New question for 2021 Census.

b Question was removed for 2021 Census.

c In the 2011 Census, this question was in reference to mental health only and, in the 2021 Census, this question asked about emotional and mental health.

The 40-year lifespan (1981–2021) of the NILS will facilitate research on intergenerational change and covers a period of historical interest known as “The Troubles.” In more recent times, Northern Ireland has experienced further societal shocks such as Brexit and COVID-19. The 2021 Census together with the linkage of additional administrative datasets permits the in-depth study of how NI has changed in both the short and longer term. The expansion of our user base to include government department researchers, co-designed projects with local councils, and charity organizations has also diversified NILS-based research, with projects focusing on how service delivery could be optimized [5].

Notably, for the latest 2021 Census, two new statistical output geographies have been designed. Data Zones and Super Data Zones were created following the public consultation on census outputs. The main reasons were to account for demographic changes such as population and housing growth/decline and to reflect areas that were becoming less socially similar over time.

## Who is in the cohort?

The NILS in 2021 had ∼515 930 members with linked census data. If someone within the centralized Northern Ireland Medical Card registration system has one of 104 pre-designated days of birth, they are included in the sample and linked to the census data. In Northern Ireland, people are entitled to “free at the point of use” health and social care, but they must be registered. Almost 100% of entitled people are contained in this medical-card registration system. Supplementary Table S1 shows a more in-depth numerical representation of the number of people in the cohort at any given time. The number of people present in all five censuses is 164 706 and the number of those who have ever been present in the NILS since 1991 is 841 601.

The NIMS comprises the whole of the census-enumerated population and subsequently registered deaths, including month, year, place, and cause of death. However, none of the additional routinely linked information is added. This differentiates this study from the NILS. There are four versions of the NIMS currently available for projects based on each of the previous four censuses (1991–2021). The NIMS is the best resource for researchers who are conducting mortality-themed research.

Tables 1–3 show the capacity of the datasets to represent even relatively small subpopulations and demonstrate that both the NILS and the NIMS cohorts are representative of official census outputs.

## What has been measured?

**Figure 1.**
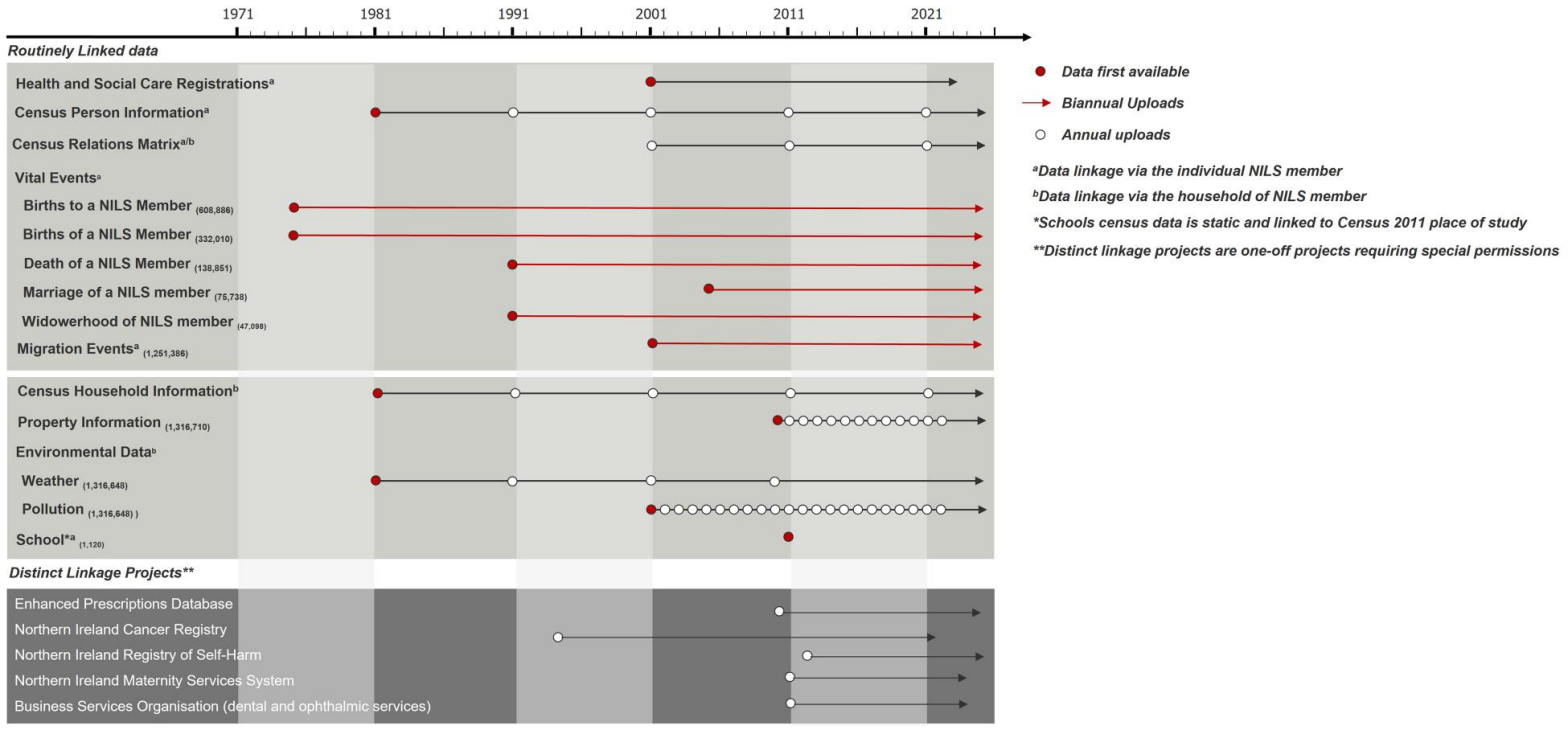
Available data within the NILS. Overview of data available within the NILS, including those that are routinely linked, and previous sources used in distinct linkage projects.

Figure 1 describes the cohort attributes. In addition to all the measures detailed in the original cohort profile [1], the three new census linkages provide information on health conditions, passports, national identity, sexual orientation, and renewable energy. School data include the type of school, an indicator of its size, special educational needs, and an indicator of students entitled to free meals, which are derived from the school census (Figure 1) and are linked to the 2011 Census by using place of study. Environmental data comprises regular updates of weather and pollution. Weather data are provided at a resolution of 5 × 5 km and are modeled by the Met Office every 10 years; pollution data are provided at a resolution of 1 × 1 km and are modeled by the Department for Environment, Food and Rural Affairs on an annual basis.

The inclusion of the Health and Care number within the NILS facilitates one-off linkages to health and social care administrative data via a Distinct Linkage Project—including hospital and laboratory systems, screening services, prescribing data (through the electronic capture of dispensed prescriptions), and uptake of dental and ophthalmic services. Northern Ireland, unlike the other parts of the UK, has combined health and social services, sharing the same unique Health and Care identifier, so it is possible to link data relating to the care of people in the community or on admission to care facilities. Cancer incidence data from 1993 to the present are held by the Northern Ireland Cancer Registry and can also be linked to the NILS. Another recently established pathway is the link to Northern Ireland Maternity Services data, allowing NILS linkage with information from a woman’s initial booking appointment at ∼12 weeks’ gestation to 7 days postpartum (Figure 1).

## What has it found? Key findings and publications

All project abstracts, their research teams, and outputs are listed on our website together with a searchable publication list. We currently have 110 projects in total, with 12 of these active as of August 2024. These are categorized under thematic areas of Ageing; Deprivation & Inequalities; Housing & Area-Based Characteristics; Medication Use; Migration & Mobility; Mortality; Psychological Behaviour & Health; Religious, Ethnic & Cultural Affiliations; Reproduction, Fertility & Pregnancy; Service Use; Work; and Health & Social Mobility. In addition, there have been several key methodological studies published by using these datasets [6, 7]. Tracking final outputs is a challenge due to the time between project completion and publication or impact achieved; however, these are monitored to the best of our ability and we have recorded 175 publications, 376 presentations, 79 papers, and 44 research/policy briefs. Additionally, at least 19 students have used the NILS or the NIMS for graduate or postgraduate research.

## What are the main strengths and weaknesses?

The NILS has a number of strengths. Its 28% sample of the Northern Ireland resident population is greater proportionally than those of the other UK census longitudinal studies. Additionally, it includes a household-relationship matrix and selected information about non-NILS household members. It holds a wide range of individual and household variables from censuses since 1981. The more recent 10-year periods between census years are bridged by regular uploads of data on important life events (births, deaths, marriages, address changes). It also has the potential for extension in the form of Distinct Linkage Projects (DLPs) as described in more detail in the “What has been measured?” section. Unlike other population-based studies, the NILS includes residents in institutionalized care. The NILS is similar to the Office of National Statistics Longitudinal Study (ONS-LS) and Scottish Longitudinal Study (SLS) in terms of structure and included variables, which has important potential for conducting cross-UK analysis [7, 9–11]. The census in 2021 had a response rate of 97%, which is the highest in recent times (92% in 2001 and 95% in 2011) [8]. The NILS has undergone significant alpha- and beta-testing processes as well as applied quality-control measures at various stages of the linkage. The overall match rate for the NILS will be slightly different for each of the iterations and will be subject to minor changes as work continues to match those who were unmatched. Further details can be found within the technical documentation, but the match rates have been >96%.

Whilst the census forms much of the strength and value of the NILS, it is also the source of some of its limitations. For those who are not enumerated, vital events and other outcomes cannot be linked to a census record and this may be further compounded by data mismatches, as there is no unique personal identifier in Northern Ireland, as in countries with a population register. The cohort attributes are also largely dependent on questions asked within the census, although the consultation on topics goes some way to mitigating this. Responses are self-reported by the individual and, if the questionnaire is completed by a self-appointed head of household, some individual information could be lost.

## Can I get hold of the data? Where can I find out more?

Due to the sensitivity of the data contained within the cohort, datasets are managed by NISRA under census legislation. The Five Safes Framework is a set of principles that has been adopted by the trusted research environment, enabling the provision of safe research access to data. The Five Safes refer to data, projects, people, settings, and outputs, and comprehensive documentation on the website outlines how the NILS adheres to each of these.

At the time of publication (February 2025), data access was possible only from within the controlled “secure environment” located in NISRA Headquarters in Colby House, Stranmillis, entry to which must be prearranged. However, access is moving towards a remote model in line with similar datasets across the UK and updates will be available on our website in the upcoming months.

The application process involves project approval by the Research Approvals Group, who will ensure that the four criteria are met: the project should have a longitudinal aspect, demonstrate how the NILS/NIMS will uniquely contribute, provide clear evidence of the value to health and social care-related research, and support the development of public policy. Furthermore, all researchers involved with a NILS project must be accredited and sign a set of license agreements to ensure compliance with statutory and regulatory rules relating to data protection and privacy before using the data. Those who access the data within the secure environment require a basic disclosure check.

Operationally, NILS–RSU is available to guide users through these processes and to provide advice and assistance to prospective users of the data. Anyone wishing to use NILS or NIMS data should visit our website (www.nils.ac.uk), where they can obtain further and more detailed information about the datasets, information on past and present projects, and a step-by-step guide through the application processes. Additionally, there is a complementary section on the NISRA website with technical guidance and documentation (https://www.nisra.gov.uk/support/research-support/northern-ireland-longitudinal-study-nils). Potential researchers are also encouraged to contact the support officers at nils@qub.ac.uk, who will help in defining a project, selecting appropriate variables, and completing a project application form. There is no cost to the user to use these services or to access the NILS.

## Ethics approval

The NILS database has ethical approval from ORECNI (REC Reference Number 22/NI/0042). However, if a potential project intends to link health data to the NILS database, further ethical approval is required. This ethical approval must be applied for by the research team.

## Supplementary Material

dyaf054_Supplementary_Data

## Data Availability

The data underlying this article were provided by NILS-RSU, NISRA by permission. Data can be accessed by an accredited researcher with permission by NILS-RSU, NISRA. Please contact corresponding author to request.
