# Supplementary material for: Cohort Profile Update: The Northern Ireland Longitudinal Study (NILS)
Source: Int J Epidemiol. 2025 May 23;54(3):dyaf054. doi: 10.1093/ije/dyaf054 (PMC12102064; doi:10.1093/ije/dyaf054)
Supplement: dyaf054_Supplementary_Data [file dyaf054_supplementary_data.docx]

**Supplementary Table S1: Number of Northern Ireland Longitudinal Study (NILS) members at each of the census years**

|  | **NILS members completing Censuses in year:** | | | | |
| --- | --- | --- | --- | --- | --- |
|  | **1981** | **1991** | **2001** | **2011** | **2021** |
| Total NILS members each Census year | Unknown | 493 673 | 504 743 | 538 612 | 573 718 |
| No. of NILS members completing: |  |  |  |  |  |
| One Census |  |  |  |  |  |
|  | 340 028 |  |  |  |  |
|  |  | 439 555 |  |  |  |
|  |  |  | 457 406 |  |  |
|  |  |  |  | 486 674 |  |
|  |  |  |  |  | 515 930 |
| Two Censuses |  |  |  |  |  |
|  | 315 586 | 315 586 |  |  |  |
|  | 266 545 |  | 266 545 |  |  |
|  | 232 379 |  |  | 232 379 |  |
|  | 197 946 |  |  |  | 197 946 |
|  |  | 356 399 | 356 399 |  |  |
|  |  | 306 160 |  | 306 160 |  |
|  |  | 267 190 |  |  | 267 190 |
|  |  |  | 373 506 | 373 506 |  |
|  |  |  | 321 449 |  | 321 449 |
|  |  |  |  | 395 704 | 395 704 |
| Three Censuses |  |  |  |  |  |
|  | 252 711 | 252 711 | 252 711 |  |  |
|  | 217 999 | 217 999 |  | 217 999 |  |
|  | 184 602 | 184 602 |  |  | 184 602 |
|  | 217 010 |  | 217 010 | 217 010 |  |
|  | 186 561 |  |  | 186 561 | 186 561 |
|  |  | 287 814 | 287 814 | 287 814 |  |
|  |  | 248 412 | 248 412 |  | 248 412 |
|  |  |  | 302 086 | 302 086 | 302 086 |
|  | 182 109 |  | 182 109 |  | 182 109 |
| Four Censuses |  |  |  |  |  |
|  | 205 641 | 205 641 | 205 641 | 205 641 |  |
|  | 174 732 | 174 732 |  | 174 732 | 174 732 |
|  | 171 924 | 171 924 | 171 924 |  | 171 924 |
|  | 174 218 |  | 174 218 | 174 218 | 174 218 |
|  |  | 233 289 | 233 289 | 233 289 | 233 289 |
| Five Censuses |  |  |  |  |  |
|  | 164 706 | 164 706 | 164 706 | 164 706 | 164 706 |
